# Medial meniscus posterior root tear reconstructed with gracilis autograft improve healing rate and patient reported outcome measures

**DOI:** 10.1186/s12891-022-06067-1

**Published:** 2022-12-14

**Authors:** Hongbo Li, Si Nie, Min Lan

**Affiliations:** 1 Department of Orthopedic Surgery, Jiangxi Provincial People’s Hospital (The First Affiliated Hospital of Nanchang Medical College), No.92 Aiguo Road, Donghu District, 330006 Nanchang, Jiangxi Province People’s Republic of China; 2 Department of Radiology, Jiangxi Provincial People’s Hospital (The First Affiliated Hospital of Nanchang Medical College), Nanchang, 330006 People’s Republic of China

**Keywords:** Medial meniscal root tear, Gracilis autograft, Transtibial pull-out repair, Arthroscopically assisted reconstruction technique, Magnetic resonance imaging

## Abstract

**Background:**

Many surgeries have not reversed or prevented progressive symptomatic knee arthritis, and there is no consensus regarding the ideal repair or reconstruction technique for meniscal root treatment. Additionally, there is a lack of studies comparing the clinical efficacy evaluation of different repair techniques. The aim of the present study is to compare the clinical efficacy and healing rates of meniscus root in the treatment of medial meniscus posterior root tear (MMPRT) with the arthroscopically assisted meniscus root reconstruction with gracilis autograft and transtibial pull-out technique.

**Methods:**

Patients with MMPRT (type II) who received treatment of posterior meniscus root attachment point through the tibial tunnel between January 2018 and April 2019 were included in this study. Patients were divided into 2 groups (arthroscopically assisted gracilis autograft reconstruction technique: 29 cases; transtibial pull-out technique group: 35 cases) according to the different treatment methods. The mean follow-up period was 26.9 ± 2.3 months. The demographics, functional recovery of the knee, and meniscus root healing rates (assessed using knee magnetic resonance imaging (MRI) at the final follow-up) were compared between the two groups.

**Results:**

There was a statistically significant improvement in the Lysholm score, international knee documentation committee (IKDC) score, and visual analogue scale (VAS) score (*P* < 0.001 in both groups). Additionally, compared with the transtibial pull-out repair group, the arthroscopically assisted reconstruction with gracilis autograft showed significant improvement in the meniscus root healing rates, Lysholm score, and IKDC score at the end of follow-up (*P* < 0.05).

**Conclusions:**

Compared with the transtibial pull-out technique, the arthroscopically assisted meniscus root reconstruction with gracilis autograft was advantageous for treating these patients with superior clinical outcome and higher meniscus root healing rates.

**Level of evidence:**

Level III.

## Introduction

The meniscus is a critical component of the knee and plays an essential role in maintaining knee functions [1]. Medial meniscus posterior root tears (MMPRTs)are relatively unfrequent and are more difficult to diagnose compared with meniscal body or horns tears; Moreover, complete posterior meniscal root tears dramatically inhibit normal meniscal function similar to total meniscectomy [2]. Studies have shown that the MMPRTs resulting in the loss of circumferential hoop stresses can change native physiologic properties of the knee, and strongly correlate with knee degeneration [3].

Therefore, studies indicated that surgical treatment should be recommended to patients with high requirements and no to low-grade osteoarthritis [4, 5]. Currently, there are several surgical options for the treatment of meniscus root tears, like meniscectomy, meniscal repair, and meniscus root reconstruction. Moreover, compared with meniscectomy or meniscal repair, some evidence indicates that meniscal root reconstruction can be performed to reestablish the normal biomechanics of the knee [5].

Transtibial pull-out repair technique by securing the meniscus posterior root to its original anatomic footprint has been reported to improve clinical outcomes [6]. However, Many repairs have not reversed or prevented progressive symptomatic knee arthritis, and there is no consensus regarding the ideal repair or reconstruction technique for meniscal root repair [6–8]. Just doing a suture on a degenerative tissue is an issue, and trying to make a degenerative meniscus heal into a tunnel is questionable. Therefore, Holmes et al. [7] present an arthroscopic reconstructive technique using gracilis autograft with suture reinforcement for MMPRT, which is expected to achieve a better healing rates compared with direct repair techniques. However, the current literature lack the clinical results of comparison between different repair techniques [8]. The purpose of this study is to compare the clinical efficacy and meniscus root healing rates in the treatment of MMPRT by the arthroscopically assisted meniscus root reconstruction with gracilis autograft and transtibial pull-out technique. Also, we hypothesize the arthroscopically assisted meniscus root reconstruction with gracilis autograft would yield better results than the transtibial pull-out technique.

## Materials and methods

### Patient selection

This study was approved by the medical research ethics committee of our institution. Diagnosis of patients with MMPRT primarily relied on clinical evidence and knee magnetic resonance imaging (MRI) findings [9] (including cleft, giraffe neck, and ghost signs). Indications for surgery included MMPRT (type II), no changes or 1–2 stages of knee osteoarthritis. Patients undergoing surgery for other indications (cartilage resurfacing, osteotomy or ligament reconstruction), previous surgery of the same knee (such as: previous tibia or femur fracture treated with surgically; osteotomy), obvious knee deformity (valgus or varus > 5°), other types of root tear, concomitant anterior cruciate ligament (ACL) injury or other associated knee joint lesions were excluded from the study.

Of the 82 MMPRT patients (type II), patients associated with ligamentous injuries (5 cases) and varus malalignment > 5° (5 cases), and concomitant high tibial osteotomy (2 cases) were excluded from the study. 70 MMPRT patients received treatment of posterior meniscus root attachment point through the tibial tunnel between January 2018 and April 2019. However, 6 patients were not evaluated because they were lost to follow-up. Ultimately, 64 MMPRT patients were included in this study. Patients were divided into 2 groups (arthroscopically assisted meniscus root reconstruction with gracilis autograft: 29 cases; transtibial pull-out technique group: 35 cases) according to the different MMPRT treatment methods. Participants were followed up for 2 years with a total of six follow-ups at directly postoperative, 1 month, 3 months, 6 months, 12 months, and 24 months.

### Data collection

The following parameters were recorded: age, gender, body mass index (BMI), comorbidities, stages OA of the knee joint evaluated according to Kellgren and Lawrence (K-L), treatment for medial meniscus posterior root tears, hospitalization time, side of injury, complications, preoperation and the final follow-up VAS (a visual analogue scale from 0 to 10 was used to assess pain), Lysholm score (the Lysholm score is a functional score designed for knee ligament injuries, which has also been validated for other knee injuries) and IKDC score of the affected knee, and radiologic outcomes of the repaired meniscus root healing status were assessed using knee MRI at the latest follow-up.

### Surgical techniques

**Fig. 1:**
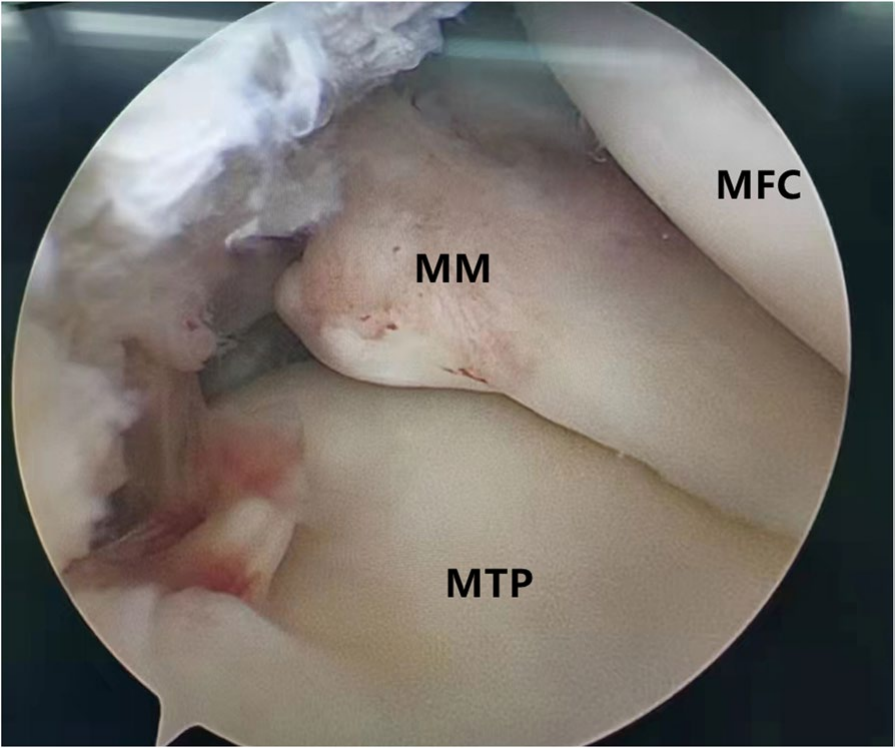
Arthroscopic evaluation and treatment of the medial meniscus posterior root tear

**Fig. 2:**
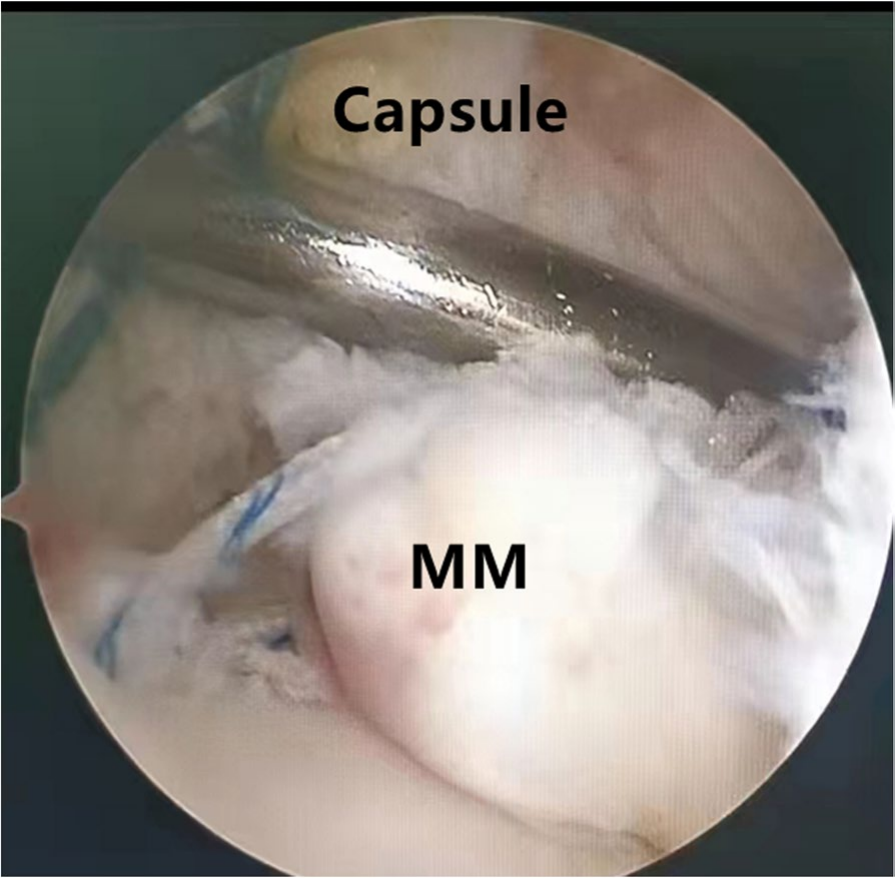
The suture shuttle was used to place a No. 0 fiber wire suture through the posterior meniscus and shuttled into the tibial tunnel and the meniscus root down into the posterior horn root attachment under arthroscopic visualization control

**Fig. 3:**
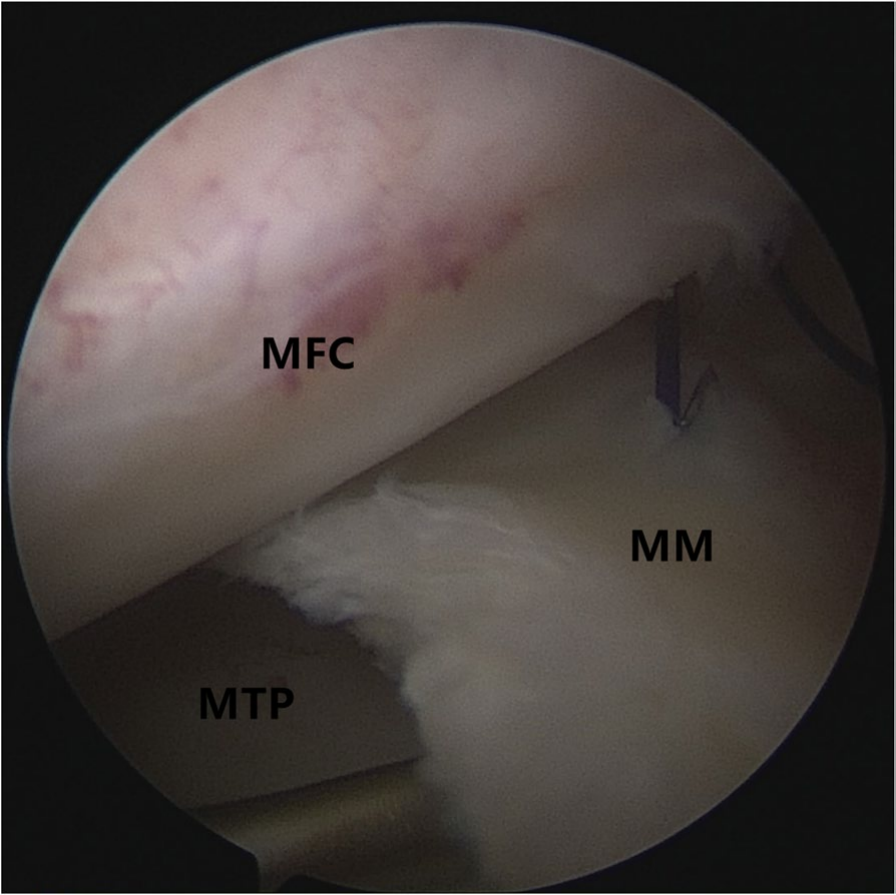
The suture shuttle was used to place a No. 0 fiber wire suture through the posterior meniscus

**Fig. 4:**
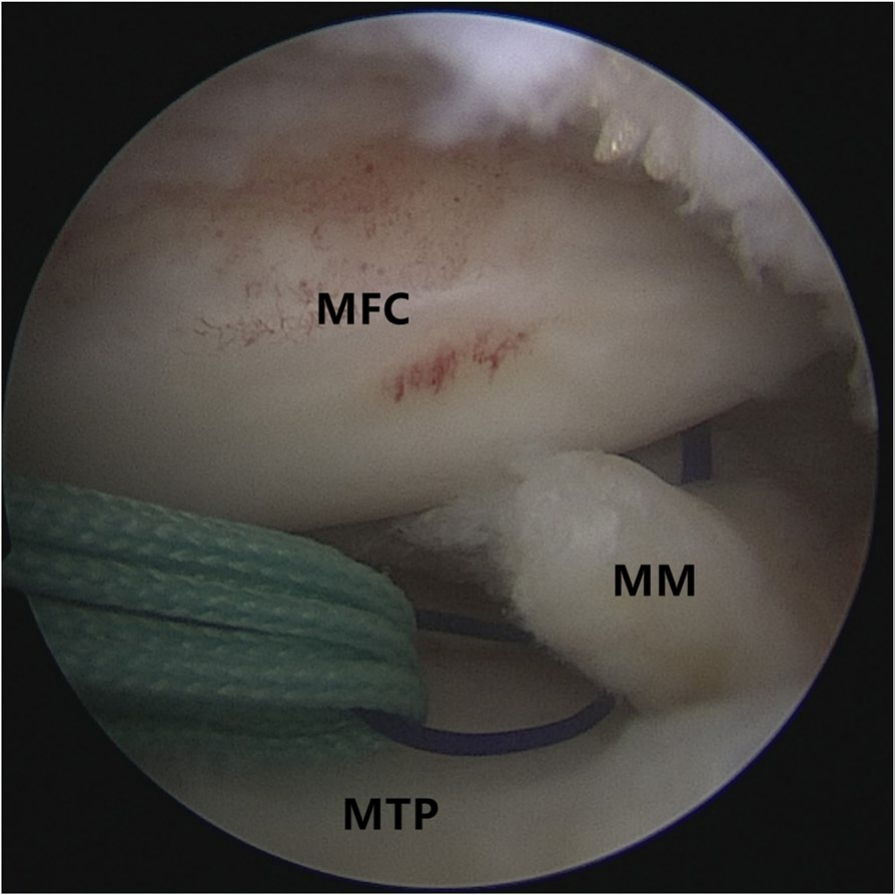
The soft tissue tunnel is dilated with multiple passes of No. 0 fiber wire

**Fig. 5:**
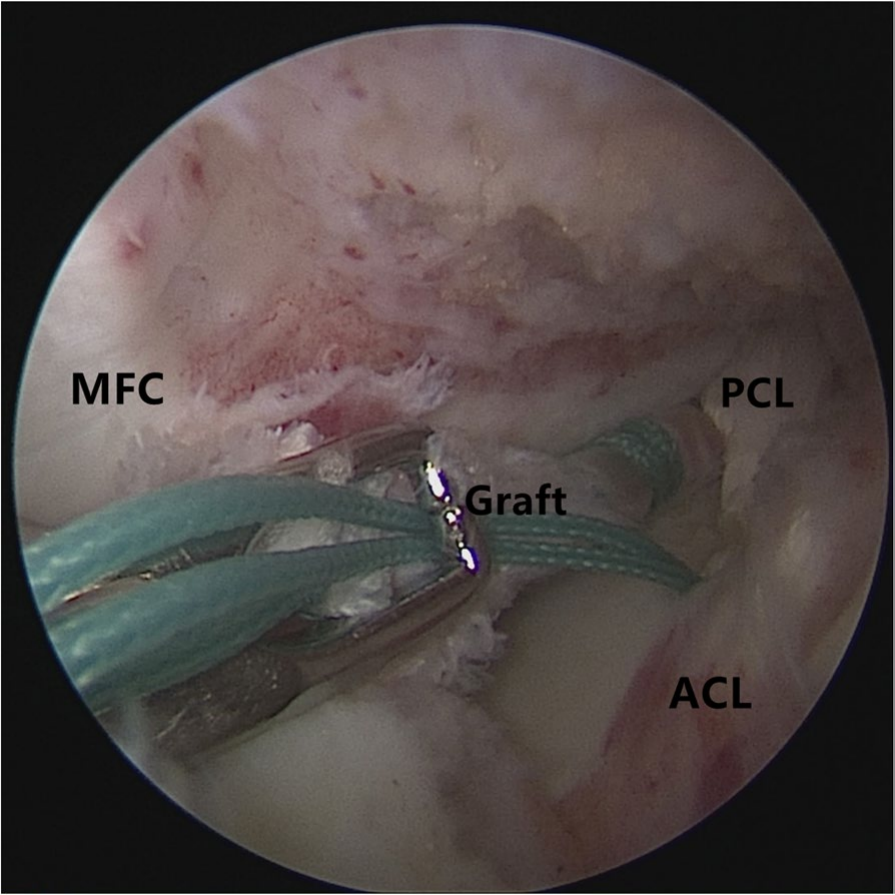
The gracilis tendon passes through the medial meniscus posterior root and shuttled into the tibial tunnel

**Fig. 6:**
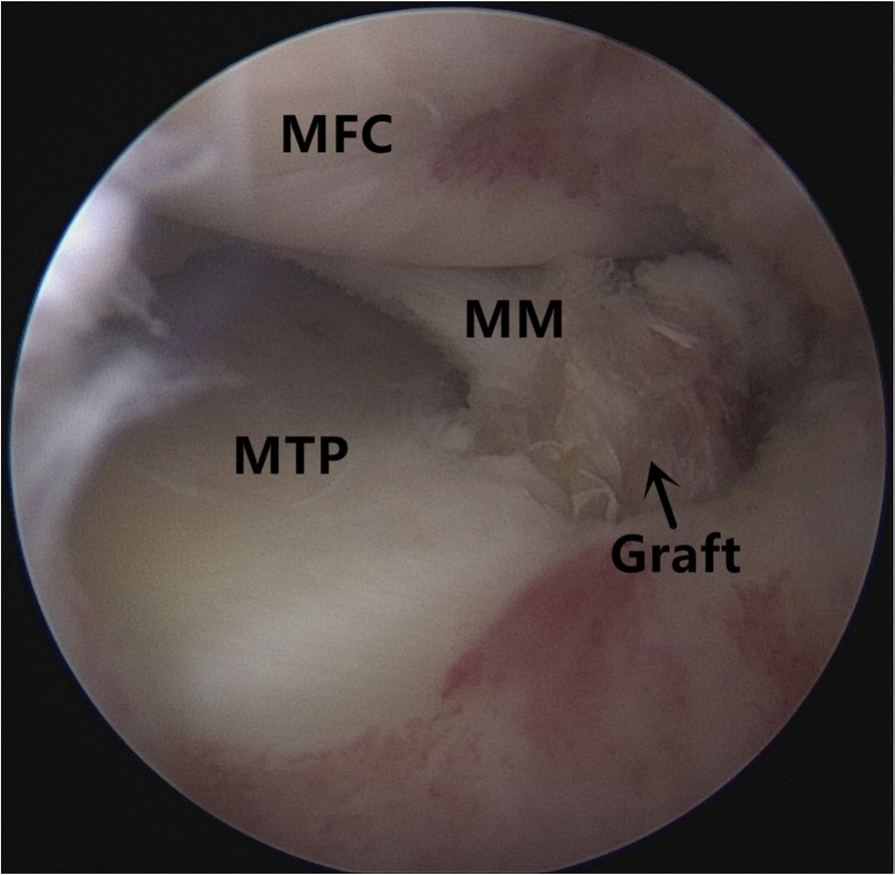
The graft has been shuttled into the tibial tunnel and placed under tension

**Fig. 7:**
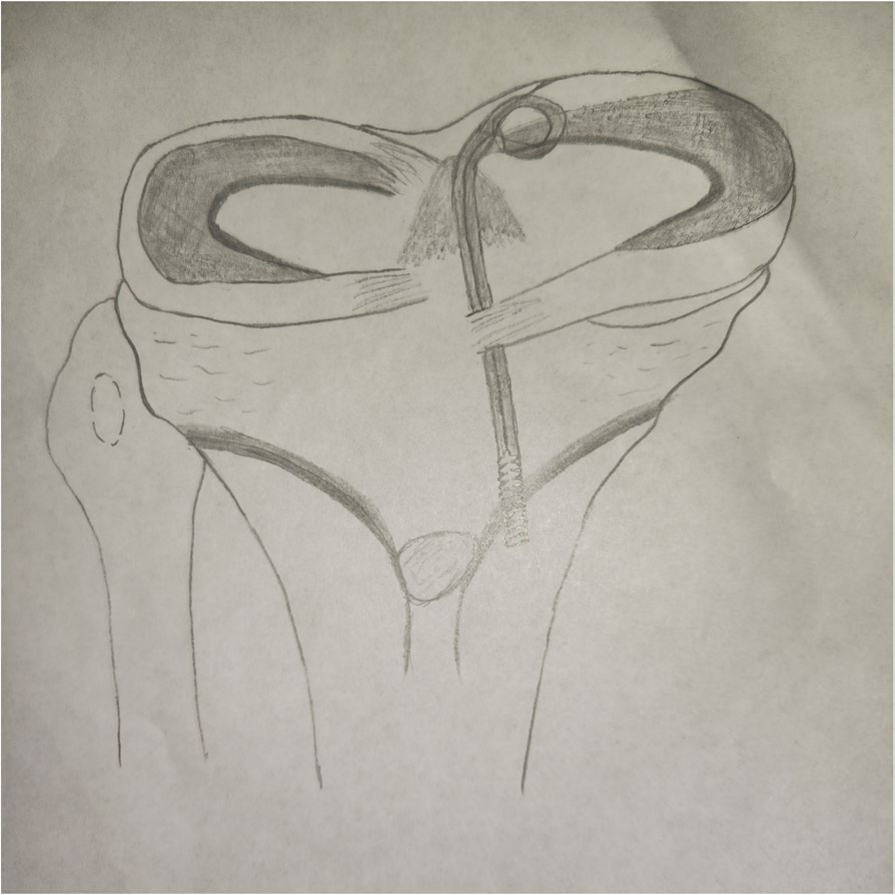
Illustration of a transtibial pull-out repair for a posterior medial meniscal root tear

Patients were placed in a supine position with knee flexion of 90°, and a pneumatic tourniquet was used after spinal anesthesia. In the pullout repair techniques group, arthroscopic evaluation of the MMPRT (Fig. 1) and other intraarticular lesions,a limited refreshment was applied to the torn edge of the meniscus with a motorized shaver, and a 2.0 mm guide pin was drilled from a small incision over the anterior proximal tibia and advanced to the posterior horn root of the knee assisted by the special guide system (Smith & Nephew, Andover, Massachusetts, USA). Then, the suture shuttle was used to place a No. 0 fiber wire suture (Smith & Nephew) through the posterior meniscus and shuttled into the tibial tunnel and the meniscus root down into the posterior horn root attachment under arthroscopic visual control (Fig. 2). The fiber wire sutures were tightened to the cortical button (Smith & Nephew, Andover, Massachusetts, USA) to ensure appropriate position and tension of the construct with knee flexion of 90°. In the arthroscopically assisted meniscus root reconstruction with gracilis autograft group, the gracilis tendon was harvested via a 2-cm longitudinal incision over the pes anserinus. The tendon was dissected and harvested with a tendon stripper. The graft was prepared and the ends were whipstitched with a No. 0 fiber wire. A 2.0 mm guide pin was drilled from a small incision over the anterior proximal tibia and advanced to the meniscus root attachment point of the knee under the special guide system (Smith & Nephew, Andover, Massachusetts, USA) assisted, then the guide pins were over-drilled with a cannulated 6-mm drill. The suture shuttle was used to place a No. 0 fiber wire suture through the posterior meniscus (Fig. 3), then the soft tissue tunnel was dilated with multiple passes of No. 0 fiber wire (Fig. 4) followed by the gracilis tendon passes through the medial meniscus posterior root (Fig. 5) and shuttled into the tibial tunnel (Fig. 6). The tails of the gracilis tendon were fixed to a 6 mm PEEK (polyether ether ketone) knotless suture anchor (Biosure RG, Smith & Nephew) to the tibial (Fig. 7), and arthroscopic visualization was used to maintain the appropriate position and tension of the graft.

### Postoperative management

Passive knee flexion and quadriceps strengthening exercises were started on the first postoperative day and were gradually increased to reach 90° of flexion after 2 weeks. Moreover, patients were allowed non-weight-bearing with two crutches for six weeks, and weight bearing was progressed as tolerated starting at 6 weeks postoperatively, a rehabilitation of 12 weeks was recommended before starting with weight-resisted exercise and half squat exercise. Return to athletic activity occurred at a minimum of 6 months postoperatively, if indicated.

### Outcome assessment

Clinical examinations were performed directly postoperative, 1 month, 3 months, 6 months, 12 months, and 24 months, and knee functional assessment was performed according to the Lysholm score, IKDC score, and VAS score. Patients and the staff collecting questionnaire data were blinded to the surgical procedure.

**Fig. 8:**
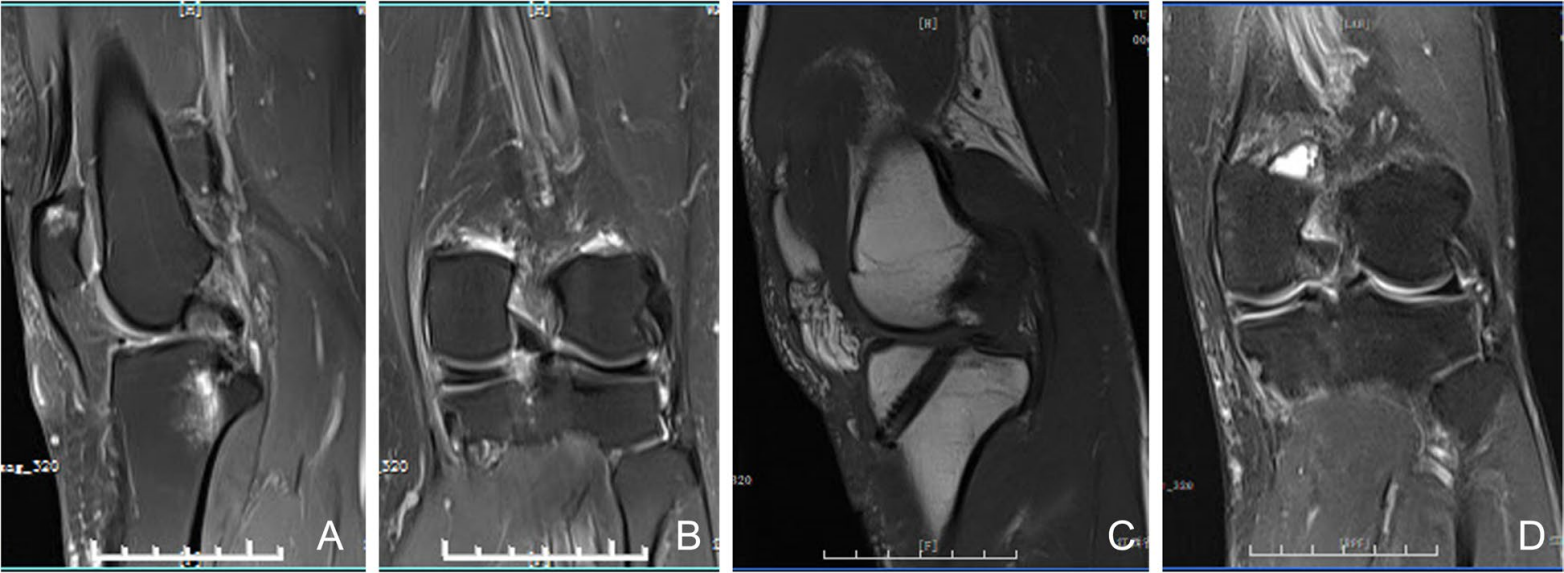
A 56-year-old male with a painful knee, **A**, **B** preoperative MRI study, Sagittal fat-suppressed proton density-weighted image (**A**) showing MMPRT radial tear with ghost signs, Coronal fat-suppressed proton density-weighted image (**B**) showing MMPRT radial tear with bone marrow edema like signal, **C**, **D** postoperative MRI study after MMPRT reconstructed with a gracilis autograft. MM: medial meniscus; MFC: medial femoral condyle; MTP: medial tibial plateau; MMPRT: medial meniscal posterior root tear

To assess the accuracy of the measurements, a second MRI reading of these factors was performed 24 months later. The imaging outcomes were evaluated by 2 trained and experienced senior orthopedic surgeons and radiologists who were blinded to pre- and postoperative status and imaging. Radiologic outcomes of the repaired meniscus root healing status were assessed using knee 3.0 T MRI (The slice thickness was 4 mm with a 0 mm gap) (Fig. 8). Meniscal root healing status was assessed according to the criteria of previous studies [10–12] and was classified as complete healing (continuity in sagittal, coronal, and axial MRI views), lax healing (loss of continuity in any 1 view), and failed healing (no continuity and no evidence of meniscal healing at the repair site).

### Statistical analysis

Quantitative variables were presented as mean value ± standard deviation (SD), and the two groups were compared using the Student’s t-test. Count variables were expressed as numbers and percentages and were assessed by the Chi-square test. Statistical significance was set as a P value less than 0.05. All analysis was performed by IBM SPSS Version 22.

## Results

### Patient demographics

As presented in Table 1, the arthroscopically assisted meniscus root reconstruction with gracilis autograft group consists of 29 subjects (mean 33.0 ± 11.2 age years), and the transtibial pull-out technique group involves 35 subjects (mean 36.1 ± 10.9 age years). The mean follow-up period is 26.9 ± 2.3 months (range, 24–32 months). In detail, there are no significant differences in age, gender, BMI, injured side, K-L grade, comorbidities, and length of postoperative hospital stay among the two groups (*P* > 0.05). Moreover, there are no significant differences in intraoperative and postoperative complications (such as stiffness, deep venous thrombosis, and infection) among the two groups (*P* > 0.05).

**Table 1:** Patient demographics in different groups

Characteristic	PT (35)	RT (29)	P
Age (y)	36.1 ± 10.9	33.0 ± 11.2	0.265
Gender: male n (%)	20 (78.6%)	17 (78.6%)	0.951
BMI (kg/m2)	21.8 ± 2.8	21.7 ± 0.7	0.881
Comorbidities			
Diabetes mellitus	3	3	1
High blood pressure	4	5	0.725
Smoking status	4	3	1
Alcohol status	5	5	1
Injured side: right n (%)	18(51.4%)	17(58.6%)	0.620
K-L			0.471
0	14	16	
I	19	12	
II	2	1	
III	0	0	
IV	0	0	
Follow-up time (months)	26.6 ± 2.2	27.4 ± 2.3	0.158
Length of postoperative hospital stay (d)	2.3 ± 0.6	2.4 ± 0.6	0.433
Postoperative complications	0	0	1
Meniscus healing rates			0.032
Complete healing	18	24	
Lax healing	10	3	
Failed healing	7	2	

*RT* arthroscopically assisted gracilis autograft reconstruction technique, *PT* transtibial pull-out technique, *K-L* Kellgren-Lawrence, *BMI* body mass index

Of the 64 root tears with postoperative MRI scans, 51.4% meniscus root tears healed completely after transtibial pull-out technique root repair. Specifically, 82.8% meniscus root tears healed completely after meniscus root reconstruction with gracilis autograft. Therefore, compared with the transtibial pull-out technique, the arthroscopically assisted meniscus root reconstruction with gracilis autograft has significant improvement in the meniscus root healing rates at the latest follow-up (*P* = 0.032).

### Comparison of functional results of the study groups

Tables 2 and 3 shows the functional recovery of the knee joint in the different groups. the postoperatively pain in the knee has been relieved in the most cases, and the Lysholm score, IKDC score, and VAS score has been significantly improved at the latest follow-up (*P* < 0.001; respectively). Furthermore, compared with the transtibial pull-out repair group, the arthroscopically assisted meniscus root reconstruction with gracilis autograft group has shown significant improvement in the Lysholm score, and IKDC score at the end of follow-up.

**Table 2:** Functional Results of the Study Groups

Characteristic	PT (35)	RT (29)	P
Preoperative VAS score	5.4 ± 1.1	5.1 ± 1.4	0.309
Preoperative Lysholm score	60.2 ± 6.2	62.0 ± 8.2	0.353
Preoperative IKDC score	61.1 ± 5.9	62.5 ± 8.0	0.437
Postoperative VAS score	1.2 ± 0.9	1.2 ± 0.8	0.996
Postoperative Lysholm score	88.0 ± 6.2	91.3 ± 3.4	0.009
Postoperative IKDC score	88.3 ± 4.7	90.7 ± 3.2	0.020

*RT* arthroscopically assisted gracilis autograft reconstruction technique, *PT* transtibial pull-out technique, *VAS* visual analogue scale, *IKDC* international knee documentation committee

**Table 3:** Functional Results of the Study Groups

Characteristic	Preoperation	Postoperative	*P*
PT group			
VAS score	5.4 ± 1.1	1.2 ± 0.9	< 0.001
Lysholm score	60.2 ± 6.2	88.0 ± 6.2	< 0.001
IKDC score	61.1 ± 5.9	88.3 ± 4.7	< 0.001
RT group			
VAS score	5.1 ± 1.4	1.2 ± 0.8	< 0.001
Lysholm score	62.0 ± 8.2	91.3 ± 3.4	< 0.001
IKDC score	62.5 ± 8.0	90.7 ± 3.2	< 0.001

*RT* arthroscopically assisted gracilis autograft reconstruction technique, *PT* transtibial pull-out technique, *VAS* visual analogue scale, *IKDC* international knee documentation committee

## Discussion

The major findings of this study are that there was a significant improvement of Lysholm score, IKDC score and VAS score postoperatively in patients who received treatment of posterior meniscus root attachment point through the tibial tunnel. Moreover, compared with the transtibial pull-out repair technique, the arthroscopically assisted meniscus root reconstruction with gracilis autograft is advantageous for treating these patients because of its superior clinical outcome and meniscus root healing rates.

Studies have shown that meniscal root tears lead to the loss of circumferential hoop stresses, and strongly correlate with progressive symptomatic joint arthritis [10]. Therefore, most of the studies have indicated that surgery should be recommended to patients with high demands and low-grade osteoarthritis [13, 14]. The transtibial pull-out technique has been reported to have promising function improvements of the knee by securing the meniscus to its original anatomic [15]. Feucht et al. [16] systematically review the outcome of the arthroscopically assisted transtibial pullout technique for MMPRT, and 62% cases with complete healing and 37% cases with lax healing have been observed at MRI or second-look arthroscopy. In present study, the postoperative knee MRI at the end of follow-up reveal that the arthroscopically assisted meniscus root reconstruction with gracilis autograft is advantageous for treating these patients with superior meniscus healing rates (51% patients achieved complete healing in the transtibial pull-out technique group vs. 82.7% patients achieved complete healing in the arthroscopically assisted gracilis autograft reconstruction technique group.).

During the past decade, growing interest has focused on the reconstruction techniques for MMPRT. Compared with the clinical and radiologic outcomes of meniscectomy or meniscal repair, there is more evidence indicating that the meniscal root reconstruction that can re-establish the native physiologic properties of the knee is beneficial for functional recovery [17, 18]. Ulku et al. [14] analyze the clinical and radiological results of arthroscopically transtibial pullout repair for the medial meniscus root, and it has been found that transtibial pull-out medial meniscus posterior root repair is an effective method for improvement of Lysholm scores (postoperative Lysholm scores are 88.8 ± 3.7) of the knee at middle follow-up period. Wang et al. [8] examine the clinical outcomes and radiological progression following MMPRT treatment, confirming MMPRT repair can not only improve functional scores (the Lysholm score has increased 28.87, IKDC score has increased 31.73), but also avoid or at least delay progressive symptomatic knee arthritis. Referring to our study, all patients enrolled in the present study have outcome scores available at 2 years or longer, and there is a statistically significant improvement in the Lysholm score, IKDC score, and VAS score at the latest follow-up.

Li et al. [19] successfully used semitendinosus tendon autograft in rabbit model, and its biomechanical properties were similar to those of the normal meniscus, demonstrating that tendon autograft is a promising alternative substitution for meniscus reconstruction. Holmes et al. [7] present compared with direct repair techniques, an arthroscopic reconstructive technique using gracilis autograft with suture reinforcement for MMPRT is expected to yield improved healing rates and clinical results. Like the ACL model, reinforced reconstruction technique require that the initial fixation be strong enough to resist initial displacement and provide a long-term stability that allows the tendon graft to heal into the meniscus and bony tunnel [18]. In consistence with previous studies, the arthroscopically assisted meniscus root reconstruction with gracilis autograft group had significant improvement in the Lysholm score, IKDC score, and meniscus healing rates at the latest follow-up in comparison with the transtibial pull-out technique group,.

Several limitations have also been detected in this study. First, our current study is a single-center study, and a relatively small number of patients may be associated with the risk of bias in the results. To address this issue, further prospective studies with increased sample size would be required to answer the question of whether the arthroscopically assisted meniscus root reconstruction with gracilis autograft is superior to the transtibial pull-out technique or not. Second, we have just observed the knee functional scores for about 2 years since medial meniscal root reconstruction, the follow-up period is relatively short and no second-look arthroscopy has been performed. Third, the main limitation of this procedure is its complexity and working in the narrow medial compartment of the knee, but it is no more complicated than commonly performed reconstructive surgery like ACL reconstruction.

## Conclusions

Compared with the transtibial pull-out technique, the arthroscopically assisted meniscus root reconstruction with gracilis autograft is advantageous for treating these patients with superior clinical outcome and meniscus root healing rates.

## Data Availability

All the data will be available upon reasonable request to the corresponding author of the present paper.

## Abbreviations

MMPRT: Medial meniscus posterior root tear
ACL: Anterior cruciate ligament injury
VAS: Visual analogue scale
MRI: Magnetic resonance imaging
K-L: Kellgren-Lawrence
IKDC: International knee documentation committee

## References

[1] Bailey L, Weldon M, Kleihege J, et al. Platelet-rich plasma augmentation of meniscal repair in the setting of anterior cruciate ligament reconstruction. Am J Sports Med. 2021;49(12):3287–92. 10.1177/03635465211036471 34477016

[2] Zhou ML, Haley CC (2021). Meniscal Ramp Lesions and Root Tears: A Review of the Current Literature. Sports Med Arthrosc Rev.

[3] Lee JI, Kim DH, Yoo HJ (2021). Comparison of the predicting performance for fate of medial meniscus posterior root tear based on treatment strategies: a comparison between logistic regression, gradient boosting, and CNN algorithms. Diagnostics (Basel).

[4] Kyun-Ho S, Hyun-Jae R, Ki-Mo J (2021). Effect of concurrent repair of medial meniscal posterior root tears during high tibial osteotomy for medial osteoarthritis during short-term follow-up: a systematic review and meta-analysis. BMC Musculoskelet Disord.

[5] Krivicich LM, Kunze KN, Parvaresh KC, et al. Comparison of long-term radiographic outcomes and rate and time for conversion to total knee arthroplasty between repair and meniscectomy for medial meniscus posterior root tears: a systematic review and meta-analysis. Sports Med Arthrosc Rev. 2022;50(7):2023–31. 10.1177/03635465211017514 34251898

[6] Krych AJ, Nauert RF, Song BM (2021). Association between transtibial meniscus root repair and rate of meniscal healing and extrusion on postoperative magnetic resonance imaging: a prospective multicenter study. Orthop J Sports Med.

[7] Holmes SW, Huff LW, Montoya KJ (2022). Arthroscopic medial meniscal root reconstruction with Gracilis autograft is safe and improves 2-year postoperative patient-reported outcomes [J]. Arthrosc Sports Med Rehabil.

[8] Wang L, Zhang K, Liu X (2021). The efficacy of meniscus posterior root tears repair: a systematic review and meta-analysis. J Orthop Surg (Hong Kong).

[9] Kintaka K, Furumatsu T, Okazaki Y (2021). Comparison of two simple stitches and modified Mason-Allen suture for medial meniscus posterior root tear based on the progression of meniscal posterior extrusion: a retrospective cohort study. J Orthop Surg (Hong Kong).

[10] Zhuo H, Chen Q, Zhu F (2020). Arthroscopic side-to-side repair for complete radial posterior lateral meniscus root tears. BMC Musculoskelet Disord.

[11] Lee OS, Lee SH, Lee YS (2021). Comparison of the radiologic, arthroscopic, and clinical outcomes between repaired versus unrepaired medial meniscus posterior horn root tear during open wedge high Tibial osteotomy. J Knee Surg.

[12] Kim SB, Ha JK, Lee SW (2011). Medial meniscus root tear refixation: comparison of clinical, radiologic, and arthroscopic findings with medial meniscectomy. Arthroscopy.

[13] Jing L, Liu K, Wang X (2020). Second-look arthroscopic findings after medial open-wedge high tibial osteotomy combined with all-inside repair of medial meniscus posterior root tears. J Orthop Surg (Hong Kong).

[14] Ulku TK, Kaya A, Kocaoglu B (2020). Suture configuration techniques have no effect on mid-term clinical outcomes of arthroscopic meniscus root repairs. Knee.

[15] Moon HS, Choi CH, Jung M (2020). Early surgical repair of medial meniscus posterior root tear minimizes the progression of meniscal extrusion: 2-year follow-up of clinical and radiographic parameters after arthroscopic transtibial pull-out repair. Am J Sports Med.

[16] Feucht MJ, Kühle J, Bode G (2015). Arthroscopic transtibial pullout repair for posterior medial meniscus root tears: a systematic review of clinical, radiographic, and second-look arthroscopic results. Arthroscopy.

[17] Lee DW, Haque R, Chung KS (2017). Arthroscopic Medial Meniscus Posterior Root Reconstruction Using Auto-Gracilis Tendon. Arthrosc Tech.

[18] Andrews SH, Rattner JB, Jamniczky HA (2015). The structural and compositional transition of the meniscal roots into the fibrocartilage of the menisci. J Anat.

[19] Li C, Hu X, Meng Q (2017). The potential of using semitendinosus tendon as autograft in rabbit meniscus reconstruction. Sci Rep.

